# Community-Based Rehabilitation (CBR) in primary care centers in Chile

**DOI:** 10.11606/s1518-8787.2020054001999

**Published:** 2020-03-30

**Authors:** Álvaro Besoain-Saldaña, Jame Rebolledo Sanhueza, Mónica Manríquez Hizaut, Valentina Cortínez Rojas, Gabriela Huepe Ortega, Verónica Aliaga-Castillo

**Affiliations:** I Universidad de Chile Facultad de Medicina Departamento de Kinesiología Santiago Chile Universidad de Chile. Facultad de Medicina. Departamento de Kinesiología. Santiago, Chile; II Universidad de Chile Programa Magíster Salud Pública Santiago Chile Universidad de Chile. Programa Magíster Salud Pública. Santiago, Chile; III Universidad de Chile Facultad de Medicina Departamento de Bioética y Humanidades Médicas Santiago Chile Universidad de Chile. Facultad de Medicina. Departamento de Bioética y Humanidades Médicas. Santiago, Chile

**Keywords:** Disabled Persons, rehabilitation, Rehabilitation centers, organization & administration, Health Plan implementation, Community participation, Health of the Disabled

## Abstract

**OBJECTIVE:**

To describe the implementation status of the Community-Based Rehabilitation in Chile.

**METHODS:**

Quantitative, transversal and descriptive study. The scope was constituted by the 66 community-based rehabilitation centers in the Chilean Metropolitan Region that implemented Community-Based Rehabilitation until December 2016. The sampling was based on a census method, so all the community centers were contacted. A self-administered questionnaire designed based on the Community-Based Rehabilitation matrix defined by the World Health Organization was applied. The questionnaire was answered on-line by the coordinators of the strategy in their respective centers. The data analysis was performed using descriptive statistics.

**RESULTS:**

A heterogeneous level of implementation of Community-Based Rehabilitation was identified, specifically in terms of the components of the matrix described by the World Health Organization. The most implemented component was Health; the Social, Livelihood and Empowerment components were moderately implemented; and the Education component was the least implemented.

**CONCLUSION:**

The implementation of Community-Based Rehabilitation is mainly based on the Health component. The level implementation of the other components of the matrix needs to be increased, as well as interdisciplinary and intersectoral strategies to achieve greater social inclusion of people with disabilities.

## INTRODUCTION

The Convention on the Rights of Persons with Disabilities (CRPD) defines that “the group of persons with disabilities includes all persons with long-term physical, mental, intellectual or sensory impairments who, in interaction with various barriers, may hinder their full and effective participation in society on an equal basis with others”^1^.

More than one billion people live with some form of impairment, which, according to 2010 population statistics, corresponds to 15% of the world’s population^2^.

**Figure 1:**
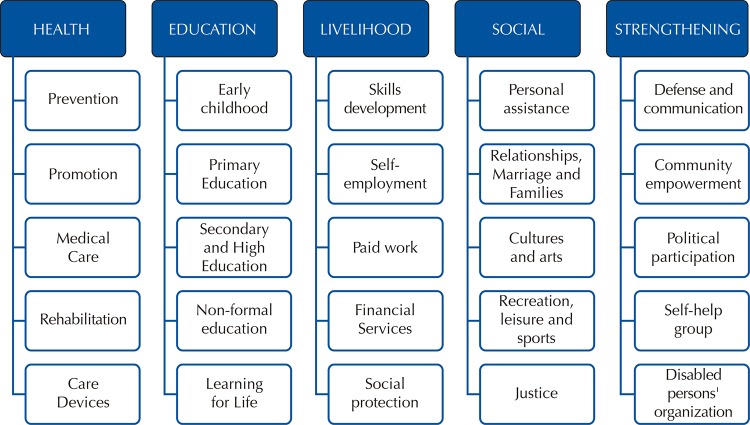
Components and elements of the CBR Matrix Source: Adapted from WHO, 2012.

The disability is associated with different factors of inequity that will lead to a situation of social, individual and familial exclusion. The main strategy defined by the World Health Organization (WHO) to achieve the reduction of these inequalities is the Community-Based Rehabilitation (CBR). This is a community development strategy for rehabilitation, equal opportunities and social inclusion, providing rehabilitation services in communities, and providing education and training opportunities to people with disabilities, their families and community members. To this end, the strategy is composed of objectives and actions in five components: health, education, work, social and community strengthening (Figure 1). Initially, CBR was seen as a strategy to improve access to rehabilitation services for people with disabilities in developing countries. However, its scope has increased significantly over the past 30 years. Today it involves local development for rehabilitation, equal opportunities, poverty reduction and social inclusion of people with disabilities^3^. This change in CBR has been associated with the paradigm change about disability and the ratification of the rights of disabled persons with the enactment of the Convention on the Rights of Persons with Disabilities^1^.

CBR is proposed as a strategy that seeks to ensure the maximum degree of social inclusion of people with disabilities and the full exercise of their rights. The reformulations converge with the principles of the Convention and are established as a multisectoral strategy that can ensure principles that make the difference in the community. While the Convention provides philosophy and policy, CBR is a practical strategy for implementation^3^. Thus, CBR activities are designed to meet the basic needs of people with disabilities and to enable access to health, education, livelihood and social opportunities^3^.

Chile began implementing CBR in 2003, incorporating it into the public health system, specifically in primary care. The purpose was to provide quality rehabilitation services in a timely and accessible way to people with permanent or transitory disabilities, improving resolution at the primary health care level^4^.

The multidisciplinary and systemic nature of CBR makes it a heterogeneous strategy, complex to implement and evaluate^5-7^. International studies about CBR in low-income countries have developed different focuses of analysis and methods. These conclude that this strategy has a positive impact on the self-esteem, empowerment, social inclusion and self-sufficiency of people with disabilities^8^. A longitudinal study of seven years of monitoring in India reports that people with disabilities who participated in CBR had more support in accessing pensions, paid work and technical aids^9^. Of the different forms of implementation, those with a bottom-up strategy, those that go from the community to the levels of local government, are best able to address the local needs of the community. However, the sustainability and solvency of the programs are vulnerable because of their dependence on volunteers and lack of stable resources^10^.

There are few studies evaluating the implementation of CBR in Chile. Some of them^11,12^show conceptual differences about international guidelines and methodological orientations of the health sector implemented in the country. The concept of community is shaped by the WHO based on belonging, interrelation and culture, forming an active and involved community. However, for the Chilean Ministry of Health, it is conceived only as a territorial unit with symbolic participation^11^. Furthermore, in Chile, CBR is mostly developed in the context of primary care centers. Funding is almost exclusively linked to the achievement of targets according to pathology care, thus diminishing the importance of other areas of the strategy. Despite these and other limitations, there is a positive evaluation in the clinical context of the functioning of the strategy^12^. Despite the change in the approach to CBR to encourage community-oriented work, the state of implementation is heterogeneous and the differences and tensions between the principles and objectives of the CBR conceptual model and the implementation process in Chile are unknown.

The objective of this study was to describe the status of CBR implementation in community rehabilitation centers in the Metropolitan Region of Chile.

## METHODS

### Quantitative, cross-sectional and descriptive study.

The census sampling method was used. The scope was defined as all community rehabilitation centers in the Chilean Metropolitan Region that implemented CBR until December 2016, and that were recognized by the National Disability Service (NDS) or by the Chilean Ministry of Health. Although there is no single official register, 66 centers were identified in the Metropolitan Region. Forty-six responses were obtained from the total number of community rehabilitation centers in the Metropolitan Region. Three centers were discarded due to inconsistencies in the information provided, resulting in a response rate of 63.6%.

Data collection was done using a self-administered questionnaire designed based on the components and elements of the CBR matrix defined by the WHO^3^ (Figure 1). The questionnaire was validated through expert judgement. Five dimensions were included: a) Profile of the team; b) Description of the activities carried out under the CBR matrix; c) Level of training of the team in the components of the CBR matrix; d) Funding strategies; and e) Community participation strategies. Likert scales and dichotomous closed questions were used. The professionals in charge of coordinating the community rehabilitation centers strategy answered the questionnaire on-line between October and December 2017. Each coordinator responded according to his/her perception of the development of each dimension during 2016.

The internal consistency of the questionnaire was established using the Cronbach alpha value overall and by dimension. A value of 0.95 was obtained on the global Cronbach alpha, which showed good internal consistency^13^. Averages and standard deviations were used for the indices that distributed normally to summarize the information of the quantitative variables. Median and interquartile ranges were used for variables that did not distribute normally. The Shapiro and Wilks test was used for tests of normality and the Pearson correlation coefficient (parametric association) and Spearman correlation coefficient (parametric association) were used for implementation indices.

An informed consent process was conducted with each coordinator. Necessary safeguards were taken to ensure the anonymity of participants and community rehabilitation centers through coding of information sources.

The Human Research Ethics Committee of the Facultad de Medicina of Universidad de Chile approved this research on December 22, 2015 (169-2015).

## RESULTS

The implementation of CBR started between 2004 and 2015. The starting point was between 2011 and 2012 (34.9%); 2.3% of the centers had more than 10 years of implementation.

Physical therapists and occupational therapists were in 100% of the teams in a proportion of 2:1 respectively. Other health professionals, such as speech speech therapists, nurses and nursing technicians, were present in less than half of the centers.

All the community rehabilitation centers conducted group rehabilitation activities, while 86.0% made home visits and individual rehabilitation activities. This was followed by participatory diagnosis activities (74.4%) and the creation of support for organizations (62.8%). The lesser activities performed were those linked to labor inclusion (41.9%) and educational integration (16.3%).

The level of implementation was estimated based on the frequency of development of activities under each of the five components of the CBR matrix described in the WHO guidelines. The most developed activities of the Health component were “Assistance Devices” (97.7%) and “Rehabilitation” (95.3%), while the less developed were “Primary Prevention” (55.8%) and “Secondary Prevention” (41.9%) (Table 1).

**Table 1:** Frequency of activities components performed Health, Education and Livelihood, Community Rehabilitation Centers, Metropolitan Region, Chile, 2016.

CBR	Activities	Category	n	%
Health	[Prevention, primary] Activities to prevent early illness, disorder or injury of DP and their families.	N / R	8	18.6
		S	11	25.6
		AA / A	24	55.8
	[Prevention, secondary] Early detection of diseases, disorders or injuries of DP and their families.	N / R	9	20.9
		S	16	37.2
		AA / A	18	41.9
	[Promotion] control of risk factors for the health of DP and their families.	N / R	5	11.6
		S	10	23.3
		AA / A	28	65.1
	[Promotion] Strengthening the skills of DP and their families to face the social, economic or environmental conditions that influence health.	N / R	8	18.6
		S	7	16.3
		AA / A	28	65.1
	[Medical Care] Timely delivery of care to identify, evaluate and/or treat health conditions of DP and their families.	N / R	1	2.3
		S	3	7.0
		AA / A	39	90.7
	[Rehabilitation] Facilitating the achievement of an optimal functioning of DP in their environment.	S	2	4.7
		AA / A	41	95.3
	[Assistive Devices] Support in the use of devices to assist DP.	S	1	2.3
		AA / A	42	97.7
Education	[Early Childhood] Supporting the inclusion of children with disabilities in preschool education.	N / R	34	79.1
		S	7	16.3
		AA / A	2	4.7
	[Primary] Support for the inclusion of children with disabilities in primary schools.	N / R	35	81.4
		S	6	14.0
		AA / A	2	4.7
	[High school] Supporting the inclusion of young people with disabilities in secondary education.	N / R	33	76.7
		S	9	20.9
		AA / A	1	2.3
	[Superior] Support for the entry and maintenance of young people with disabilities in higher education.	N / R	34	79.1
		S	7	16.3
		AA / A	2	4.7
	[Non-formal education] Non-formal educational activities for children and young people with disabilities in the community.	N / R	36	83.7
		S	7	16.3
	[Lifelong learning] Learning activities for DP who have not been covered by formal education.	N / R	24	55.8
		S	16	37.2
		AA / A	3	7.0
Livelihood	[Basic Skills Development] Work skills (literacy, math or learning skills).	N / R	21	48.8
		S	14	32.6
		AA / A	8	18.6
	[Technical Skills Development] Technical and professional skills (carpentry, shoemaking, weaving, craftsmanship).	N / R	21	48.8
		S	10	23.3
		AA / A	12	27.9
	[Entrepreneurship Skills Development] Skills for entrepreneurship (administration, planning or working with people).	N / R	24	55.8
		R	11	25.6
		AA / A	8	18.6
	[Skills Development] Life skills (teamwork, interpersonal skills, creative thinking.	N / R	14	32.6
		S	12	27.9
		AA / A	17	39.5
	[Self-employment] Support for the development of self-employed income-generating activities.	N / R	20	46.5
		S	11	25.6
		AA / A	12	27.9
	[Paid work] Activities to overcome and/or reduce barriers to entry into paid work.	N / R	22	51.2
		S	13	30.2
		AA / A	8	18.6
	[Social protection, social benefits] Promoting access to social benefits provided by the State or other organizations.	N / R	9	20.9
		S	12	27.9
		AA / A	22	51.2
	[Social Protection, support networks] Development of social support networks (self-help groups, community organizations, family support).	N / R	6	14.0
		S	6	14.0
		AA / A	31	72.1

Source: Our elaboration.

N= never, R= rarely, S= sometimes, AA= almost always, A= always, DP= disabled person

The level of implementation of all activities in the Education component was low, with the activity “Lifelong Learning” standing out at 7%, in contrast to the absence of implementation of “Non-formal Education” for children and young people (Table 1).

The activities “Social protection, support networks” were the most implemented in the Education component with 72.1%, while “Basic skills development,” “Entrepreneurship skills development” and “Paid work” were implemented with 18.6% each one (Table 1).

On the other hand, most of the activities of the Social and Strengthening components had high levels of implementation (Table 2).

**Table 2:** Frequency of activities performed components Social and Strengthening. Community Rehabilitation Centers, Metropolitan Region, Chile, 2016.

CBR	Questions	Category	n	%
Social	[Personal Assistance] Support access, administration of non-family personal assistance necessary for living with self-determination and dignity.	N / R	12	27.9
		S	16	37.2
		AA/A	15	34.9
	[Maternity and Paternity] Facilitate access to services and programs to support DP in their maternity and paternity.	N / R	28	65.1
		S	7	16.3
		AA/A	8	18.6
	[Relationships, marriage and family] Support for the development of satisfactory family relationships.	N / R	7	16.3
		S	10	23.3
		AA/A	26	60.5
	[Social Relations] Encouragement and support for DP to socialize and develop relationships outside home.	N / R	2	4.7
		S	7	16.3
		AA/A	34	79.1
	[Relationships, marriage and family] Raising awareness about disability, preventing associated violence and changing negative attitudes of both family members and the community.	N / R	5	11.6
		S	5	11.6
		AA/A	33	76.7
	[Culture and the arts] Activities that help develop the identity of DP.	N / R	7	16.3
		S	6	14
		AA/A	30	69.8
	[Culture and arts] Developing the sense of belonging of the DP to his/her community and territory	N / R	9	20.9
		S	8	18.6
		AA/A	26	60.5
	[Culture and arts] Supporting access and inclusion of DP to cultural and artistic activities	N / R	11	25.6
		S	9	20.9
		AA/A	23	53.5
	[Recreation, leisure and sports] Promoting the participation of DP in recreation and sports activities.	N / R	5	11.6
		S	12	27.9
		AA/A	26	60.5
	[Justice] Promoting the DP and family members to use community and/or judicial support when their rights are violated.	N / R	10	23.3
		S	16	37.2
		AA/A	17	39.5
Strengthening	[Defense and Communication] Encouraging the use of technology or support to ensure the accessibility of information and communication.	N / R	14	32.6
		S	17	39.5
		AA/A	12	27.9
	[Defense and Representation] Support for DP and their families to represent themselves in their respective communities.	N / R	12	27.9
		S	18	41.9
		AA/A	13	30.2
	[Community Empowerment] Encourage the community to improve the quality of life of DP and their families.	N / R	8	18.6
		S	9	20.9
		AA/A	26	60.5
	[Political Participation] Promote the participation of DP in formal politics (political decision-making and local or central development programmes).	N / R	15	34.9
		S	12	27.9
		AA/A	16	37.2
	[Political Participation Skills] Supporting DP and their families in accessing information and developing skills to participate in politics.	N / R	22	51.2
		S	12	27.9
		AA/A	9	20.9
	[Self-Help Groups] Encouraging self-help groups for common activities and problem solving.	N / R	13	30.2
		S	9	20.9
		AA/A	21	48.8
	[Formation of DP organizations] Support the formation of DP organizations.	N / R	11	25.6
		S	9	20.9
		AA/A	23	53.5

Source: Our elaboration.

N= never, R= rarely, S= sometimes, AA= almost always, A= always, DP= disabled person

The “Social Relations” activity had the highest percentage of implementation (79.1%) of the Social component versus the “Maternity and Paternity” activity with 18.6%.

The most developed activity of the Strengthening component was “Community empowerment” (60.5%) in contrast to the activity “Skills for political participation” (20.9%).

**Figure 2:**
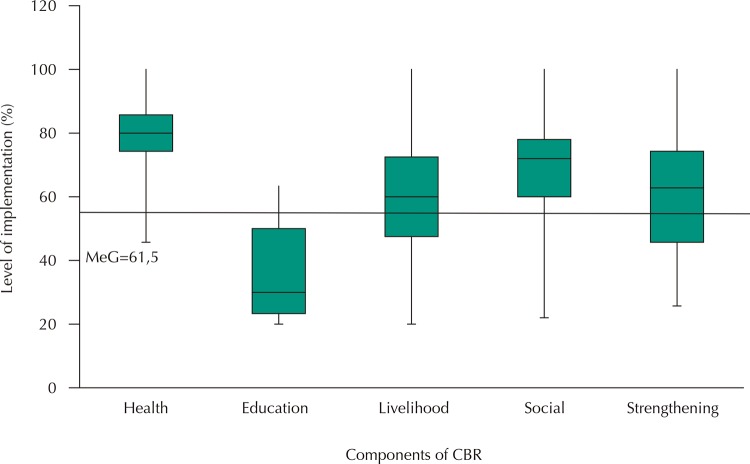
Rate of implementation by CBR component. MeG (acronym in Spanish) = Global overall

Source: Our elaboration

An implementation index was developed based on the results of the activities of each component (Figure 2). A 100% level of implementation was assumed for a component in which responses to all activities were “Always” and 0% when responses to all activities were “Never.” Based on this index, an overall level of implementation of 61.5% was described, with a range of 26.7% to 92.0%. The Education component had a lower level of implementation, with a median of 30.0%, and a range of 20.0% to 63.3%, while the Health component had a higher level of implementation with a median of 80.0% and a range of 45.7% to 100%. The Social, Strengthening, and Livelihood components showed a wide dispersion of data (varying between 20.0% and 100% implementation) and with median values of 60, 72, and 62.9, respectively. A correlation of this index was identified between all components, except between Education and Health (Spearman’s rho 0.298; p = 0.06), while the highest levels of association at the level of implementation were between the Strengthening and Social indexes (Spearman’s rho 0.693; p < 0.001) and Health and Livelihood (Spearman’s rho 0.614; p < 0.001).

When comparing the level of global implementation and by component of the activities performed in the centers, those that declare to perform “Training of organizations of people with disabilities” presented a higher level of implementation of the CBR matrix than those that do not declare to perform them (p < 0.001 between each of the components).

The teams had different levels of training in the components of the CBR matrix. 65.1% of the centers reported a “very high” and “high” level of training in the Health component, while 62.8% of the responses in the Education component were distributed between “low” and “very low” levels of training (Table 3).

**Table 3:** Level perception of professional team training in the components of CBR. Community Rehabilitation Centers, Metropolitan Region, Chile, 2016.

Perception of the overall level of training		n	%
in health	Very Low	1	2.3
	Low	1	2.3
	Medium	13	30.2
	High	20	46.5
	Very high	8	18.6
in education	Very Low	9	20.9
	Low	18	41.9
	Medium	14	32.6
	High	2	4.7
	Very high	0	0.0
in livelihood	Very Low	5	11.6
	Low	7	16.3
	Medium	20	46.5
	High	8	18.6
	Very high	3	7.0
in the social sphere	Very Low	3	7.0
	Low	11	25.6
	Medium	17	39.5
	High	8	18.6
	Very high	4	9.3
in strengthening	Very Low	3	7.0
	Low	11	25.6
	Medium	18	41.9
	High	5	11.6
	Very high	6	14.0

Source: Our elaboration.

CBR = Community-Based Rehabilitation

The main sources of funding for CBR were the Ministry of Health (86.0%) and local governments (65.1%). Other sources of funding were also reported, such as self-managed funds (16.3%) and funds provided by the National Disability Service (7.0%). Only one center reported receiving funding from private sources.

The community rehabilitation centers were linked to community organizations and institutions in the local level territory. The community and local level organizations with the greatest links were the municipal local government (51.2%), the neighborhood organizations (37.2%), the Municipal Office for Disability and Health Centers (both with 34.9%). On the other hand, central government (14.0%), educational centers (14.0%), health user organizations (14.0%), foundations, corporations and non-governmental organizations (11.6%), Municipal Office of Labor Mediation (11.6%), organizations of persons with disabilities (7.0%) and the private sector (4.7%), were the bodies with less involvement reported.

## DISCUSSION

This study is one of the first approaches to evaluate the implementation of CBR in Latin America after the ratification of CRPD.

A heterogeneous state of the implementation of CBR was identified in community-based rehabilitation centers in the Metropolitan Region of Chile, specifically with regard to the components of the matrix defined by WHO. The implementation of the strategy focuses on the “Health” component. This component is the most present in the usual practices of medical care, rehabilitation and support in the use of assistive devices and where a higher level of training of professionals is reported. In contrast, the “Education” component reports the lowest level of implementation and training of professionals. This is consistent with the fact that the activities and indicators of the Chilean Ministry of Health’s community-based rehabilitation programme focus on physical rehabilitation and on adults as the target population^14^.

The main objective of the CBR strategy is to ensure the maximum degree of social inclusion and exercise of rights by people with disabilities^3^. In Chile, the goal of the CBR program is that 10% of the people served in community rehabilitation centers achieve social inclusion (incorporation into work, school, social groups or organizations)^4^. However, most centers report few activities in the Education and Livelihood components, such as educational leveling and job skills development activities. This represents a barrier to the achievement of the proposed objectives in the area of social inclusion and the rights exercise. This finding coincides with other studies that show the incorporation of socio-economic supports, such as labor intermediation or vocational training, in addition to physical rehabilitation; contribute to inclusion, to improving people’s self-esteem and to changing society’s attitude towards them^15^.

All centers have physical therapists and occupational therapists, and less than half have professionals from other disciplines, such as speech therapists, nurses, psychologists and nursing technicians. Although the existence of the dual physical-occupational therapist is an advance in the implementation of a comprehensive rehabilitation model, it is still insufficient for the adequate implementation of the CBR strategy. In addition, there is little intersectoral work. 51.2% of the centers report having links with local government in the context of the Metropolitan Region. In addition, there is little involvement of other partners, such as monitors and community agents, to promote the implementation of primary and secondary prevention activities and other activities related to mental health, education and community strengthening. This finding coincides with evaluation studies on CBR programmes in other countries (Malawi, Kenya, Tanzania and Uganda) which indicate that the limited human resources and training is a major constraint to their implementation^16^.

If we hope to achieve “no one left behind, through the full implementation of the Convention on the Rights of Persons with Disabilities,” as established by the CRPD monitoring committee in June 2018, we must position CBR as a trans-sectoral strategy that achieves social inclusion. Continuing to focus only on reducing the gap in access to rehabilitation will not provide either the tools or the results required for sustainable development to be achieved at 2030.

It is recognized as limitations of this study that the information was obtained from CBR coordinators. This creates the possibility of overestimating the state of implementation by reporting on their own performance as managers. However, the perspective of the agents responsible for the community rehabilitation centers is required, since in their coordinating role, they have great importance in the implementation of the programme. Future research should include the professional team and people with disabilities and their families in the evaluation processes of the community centers in order to deepen and capture the complexity of the implementation. This would allow for compliance with the principles of CRPD and a comprehensive evaluation of the activities implemented.

Furthermore, it is only possible to refer to the state of implementation of CBR in the Metropolitan Region and not to the entire national territory, given the geographical and socio-demographic characteristics of the region as the capital of the country. On the other hand, there was no instrument to evaluate the activities conducted in the context of CBR implementation in Chile. From this study, an instrument is available allowing the collection of complete information about the implementation of CBR in all the national territory.

The implementation of CBR in Chile is focused on the health component of the matrix proposed by the WHO. This fact must be considered in the processes of evaluation and formulation of plans to improve the strategy. Not only is it important to continue with the implementation of the other components of the matrix, but also to complete the implementation of the Health component. This should be done through the incorporation of promotion, prevention, support for the use of technical aids and comprehensive care that includes attention in the field of mental health to accompany the processes of acceptance and adaptation, both for persons with disabilities and their families.

The strategy should enhance intersectoral work and recognize institutional and community members, with whom they should generate alliances to join efforts to build a more inclusive society. CBR, even in health-focused programmes, can promote positive coexistence between models of understanding about disability (medical and social). This is provided that rehabilitation services respond to the needs and interests of people with disabilities and that there are opportunities for training and reflection concerning society’s role in establishing barriers to inclusion. It is necessary to have a greater diversity of professionals in order to expand the activities implemented in CBR centers. On the other hand, it is essential to support the development of organizations of people with disabilities to defend their rights and thus advance in the construction of a more fair and inclusive society.
