# Supplementary material for: Community-Based Rehabilitation (CBR) in primary care centers in Chile
Source: Rev Saude Publica. 2020 Mar 30;54:38. doi: 10.11606/s1518-8787.2020054001999 (PMC7112743; doi:10.11606/s1518-8787.2020054001999)
Supplement: Translation

# Translation: Rehabilitación Basada en la Comunidad (RBC) en centros de atención primaria en Chile

**Authors:** Álvaro Besoain-Saldaña, Jame Rebolledo Sanhueza, Mónica Manríquez Hizaut, Valentina Cortínez Rojas, Gabriela Huepe Ortega, Verónica Aliaga-Castillo

**Affiliations:** I Chile Universidad de Chile, Facultad de Medicina, Departamento de Kinesiología, Santiago, Chile; II Chile Universidad de Chile, Programa Magíster Salud Pública, Santiago, Chile; III Chile Universidad de Chile, Facultad de Medicina, Departamento de Bioética y Humanidades Médicas, Santiago, Chile

**Keywords:** Personas con Discapacidad, rehabilitación, Centros de Rehabilitación, organización & administración, Implementación de Plan de Salud, Participación de la Comunidad, Salud de la Persona con Discapacidad

## Abstract

**OBJETIVO:**

Describir el estado de implementación de la Rehabilitación Basada en la Comunidad en Chile.

**MÉTODOS:**

Estudio cuantitativo, transversal y descriptivo. El universo estuvo conformado por los 66 centros comunitarios de rehabilitación de la Región Metropolitana de Chile que implementaron la Rehabilitación Basada en la Comunidad hasta diciembre de 2016. El muestreo tuvo intención censal, por lo que se contactaron todos los centros comunitarios. Se aplicó un cuestionario autoadministrado diseñado con base a la matriz de Rehabilitación Basada en la Comunidad definida por la Organización Mundial de la Salud. El cuestionario fue respondido *on-line* por los coordinadores de la estrategia en sus respectivos centros. El análisis de datos se realizó utilizando estadígrafos descriptivos.

**RESULTADOS:**

Se identificó un nivel heterogéneo de implementación de la Rehabilitación Basada en la Comunidad, específicamente en cuanto a los componentes de la matriz descrita por la Organización Mundial de la Salud. El componente más implementado fue Salud; los componentes Social, Subsistencia y Fortalecimiento fueron medianamente implementados; y el componente Educación fue el menos implementado.

**CONCLUSIÓN:**

La implementación de la Rehabilitación Basada en la Comunidad se basa principalmente en el componente Salud. Se requiere aumentar el nivel de implementación de los otros componentes de la matriz, así como de estrategias interdisciplinarias e intersectoriales para lograr una mayor inclusión social de las personas con discapacidad.

## INTRODUCCIÓN

La Convención sobre los Derechos de las Personas con Discapacidad (CDPD) define que “dentro del grupo de las personas con discapacidad se incluyen a todas aquellas que tengan deficiencias físicas, mentales, intelectuales o sensoriales a largo plazo que, al interactuar con diversas barreras, puedan impedir su participación plena y efectiva en la sociedad, en igualdad de condiciones con las demás”^1^.

Más de mil millones de personas viven con algún tipo de discapacidad, lo que significa, de acuerdo a las estadísticas poblacionales de 2010, un 15% de la población mundial^2^.

La discapacidad se asocia a diferentes factores de inequidad que conducirán a una situación de exclusión social individual y familiar. La principal estrategia definida por la Organización Mundial de la Salud (OMS) para lograr la reducción de estas inequidades es la Rehabilitación Basada en la Comunidad (RBC). Esta es una estrategia de desarrollo comunitario para la rehabilitación, la igualdad de oportunidades y la inclusión social, prestando servicios de rehabilitación en las comunidades, y proveyendo de educación y oportunidades de entrenamiento y capacitación a las personas con discapacidad, sus familiares y miembros de la comunidad. Para esto, la estrategia se compone de objetivos y acciones en cinco componentes: salud, educación, trabajo, social y fortalecimiento comunitario (Figura 1). En un comienzo, la RBC fue considerada una estrategia que mejoraba el acceso a los servicios de rehabilitación para las personas con discapacidad en los países en vías de desarrollo. Sin embargo, su alcance se ha incrementado de forma importante en los últimos 30 años. Hoy involucra el desarrollo local para la rehabilitación, la igualdad de oportunidades, la disminución de la pobreza y la inclusión social de las personas con discapacidad^3^. Este giro en la RBC se ha asociado al cambio de paradigma sobre la discapacidad y a la ratificación de los derechos de las personas con discapacidad con la promulgación de la Convención sobre los Derechos de las Personas con Discapacidad^1^.

**Figura 1:** Componentes y elementos de la Matriz de RBC. Fuente: Adaptada de OMS, 2012.

La RBC es propuesta como una estrategia que busca asegurar el máximo grado de inclusión social de las personas con discapacidad y el pleno ejercicio de sus derechos. Las reformulaciones confluyen con los principios de la Convención y se establece como una estrategia multisectorial que puede asegurar que estos principios hagan una diferencia en la comunidad. Mientras la Convención brinda filosofía y política, la RBC es una estrategia práctica para su implementación^3^. En ese sentido, las actividades de la RBC se diseñaron para satisfacer las necesidades básicas de las personas con discapacidad y permitir el acceso a salud, educación, subsistencia y oportunidades sociales^3^.

Chile inició la implementación de la RBC en el 2003, incorporándose en el sistema público de salud, específicamente en la atención primaria. El propósito fue entregar prestaciones de rehabilitación de calidad de forma oportuna y accesible a las personas en situación de discapacidad, permanente o transitoria, mejorando la resolutividad a nivel de atención primaria de salud^4^.

El carácter multidisciplinario y sistémico de la RBC hace de ella una estrategia heterogénea, compleja de implementar y evaluar^5-7^. Estudios internacionales sobre RBC realizados en países de bajos ingresos han desarrollado distintos focos de análisis y métodos. Concluyen que esta estrategia impacta positivamente en la autoestima, el empoderamiento, la inclusión social y autosuficiencia de las personas con discapacidad^8^. Un estudio longitudinal de siete años de seguimiento en India da cuenta de que las personas con discapacidad que participaron de la RBC tuvieron más apoyos para el acceso a pensiones, trabajo remunerado y ayudas técnicas^9^. De las diferentes formas de implementación, aquellas en las cuales la estrategia es ascendente, es decir, desde la comunidad a los niveles de gobierno local, logra abordar de mejor forma las necesidades locales de la comunidad. Sin embargo, se ven amenazadas la sostenibilidad y la solvencia de los programas por depender de voluntarios y no tener recursos estables^10^.

Se han realizado escasos estudios que evalúen la implementación de la RBC en Chile. Algunos de ellos^11,12^ muestran diferencias conceptuales sobre los lineamientos internacionales y las orientaciones metodológicas del sector salud implementadas en el país. La OMS configura el concepto de comunidad desde la pertenencia, interrelación y cultura, configurándose una comunidad activa e involucrada. Sin embargo, para el Ministerio de Salud chileno, se concibe solo como una unidad territorial con participación simbólica^11^. Además, en Chile, la RBC se desarrolla mayoritariamente en el contexto de los centros de atención primaria. El financiamiento está vinculado casi exclusivamente al logro de metas según atenciones por patologías, restándole así importancia a otras áreas de la estrategia. A pesar de estas y otras limitaciones, existe una positiva evaluación en el ámbito clínico respecto al funcionamiento de la estrategia^12^. A pesar del giro planteado en la RBC con el propósito de incentivar su trabajo con orientación comunal, el estado de implementación es heterogéneo y se desconocen las diferencias y tensiones existentes entre los principios y objetivos declarados en el modelo conceptual de la RBC y el proceso de implementación en Chile.

El objetivo de este estudio fue describir el estado de implementación de la RBC en centros comunitarios de rehabilitación de la Región Metropolitana de Chile.

## MÉTODOS

### Estudio cuantitativo, transversal y descriptivo.

Se utilizó un muestreo con intención censal. Se definió como universo a todos los centros comunitarios de rehabilitación de la Región Metropolitana de Chile que implementaron la RBC hasta diciembre de 2016 y que tuvieran el reconocimiento del Servicio Nacional de la Discapacidad (SENADIS) o del Ministerio de Salud chileno. Si bien no existe un registro oficial único, se han reconocido 66 centros en la Región Metropolitana. Se obtuvieron 46 respuestas del total de centros de rehabilitación comunitaria de la Región Metropolitana. Se descartaron tres por inconsistencia de la información entregada, consiguiendo un nivel de respuesta del 63,6%.

La recolección de datos se realizó mediante un cuestionario autoadministrado diseñado con base en los componentes y elementos de la matriz de RBC definida por la OMS^3^ (Figura 1). El cuestionario fue validado a través de juicio de experto. Se incluyeron cinco dimensiones: a) Perfil del equipo de trabajo, b) Descripción de las actividades realizadas en el marco de la matriz de RBC, c) Nivel de capacitación del equipo en los componentes de la matriz de RBC, d) Estrategias de financiamiento y e) Estrategias de participación comunitaria. Se utilizaron escalas Likert y preguntas cerradas dicotómicas. El cuestionario fue respondido *on-line* entre octubre y diciembre de 2017 por los profesionales a cargo de la coordinación de la estrategia de los centros comunitarios de rehabilitación. Cada coordinador(a) respondió de acuerdo a su percepción sobre el desarrollo de cada dimensión durante el año 2016.

Se estableció la consistencia interna del cuestionario mediante el valor alfa de Cronbach global y por dimensión. Se obtuvo un valor de 0,95 en el alfa de Cronbach global, lo que mostró una buena consistencia interna^13^. Se utilizaron promedios y desviaciones estándar para los índices que distribuyeron normal para resumir la información de las variables cuantitativas. Se utilizaron mediana y rangos intercuartílicos para variables que no distribuyeron normal. Se utilizó el test de Shapiro y Wilks para pruebas de normalidad y el coeficiente de correlación de Pearson (asociación paramétrica) y coeficiente de correlación de Spearman (asociación no paramétrica) para los índices de implementación.

Se realizó un proceso de consentimiento informado con cada coordinador(a). Se tomaron los resguardos necesarios para garantizar el anonimato de los participantes y de los centros comunitarios de rehabilitación a través de la codificación de las fuentes de información.

Esta investigación fue aprobada por el Comité de Ética de Investigación en Seres Humanos de la Facultad de Medicina de la Universidad de Chile el 22 de diciembre de 2015 (169-2015).

## RESULTADOS

La implementación de la RBC se inició entre los años 2004 y 2015. La moda de inicio se encontró entre los años 2011 y 2012 (34,9%). Un 2,3% de los centros tenían más de 10 años de implementación.

En el 100% de los equipos había kinesiólogos/fisioterapeutas y terapeutas ocupacionales en una proporción 2:1 respectivamente. Otros profesionales sanitarios, tales como fonoaudiólogos/logopedas, enfermeras(os) y técnicos de nivel superior, estuvieron presentes en menos de la mitad de los centros.

El 100% de los centros de rehabilitación comunitaria realizaba actividades de rehabilitación grupales, mientras que un 86,0% realizaba visitas domiciliarias y actividades de rehabilitación individual. Le siguen las actividades de diagnóstico participativo (74,4%) y de creación de apoyo a organizaciones (62,8%). Las actividades menos realizadas fueron las vinculadas a inclusión laboral (41,9%) e integración educacional (16,3%).

Se estimó el nivel de implementación basado en la frecuencia de desarrollo de las actividades propias de cada uno de los cinco componentes de la matriz de la RBC descrita en las guías de la OMS. Las actividades del componente Salud más desarrolladas fueron “Dispositivos de Asistencia” (97,7%) y “Rehabilitación” (95,3%), mientras que las menos desarrolladas fueron “Prevención primaria” (55,8%) y “Prevención secundaria” (41,9%) (Tabla 1).

**Tabla 1:** Frecuencia de actividades realizadas componentes Salud, Educación y Subsistencia, Centros Comunitarios de Rehabilitación, Región Metropolitana, Chile, 2016.

RBC	Actividades	Categoría	n	%
Salud	[Prevención, primaria] Actividades para evitar de manera temprana enfermedades, desórdenes o lesiones de las PcD y sus familias	N / MPV	8	18,6
		AV	11	25,6
		CS / S	24	55,8
	[Prevención, secundaria] Detección temprana de enfermedades, desórdenes o lesiones de PcD y sus familias	N / MPV	9	20,9
		AV	16	37,2
		CS / S	18	41,9
	[Promoción] control de factores de riesgo para la salud de PcD y sus familias.	N / MPV	5	11,6
		AV	10	23,3
		CS / S	28	65,1
	[Promoción] Fortalecimiento de las destrezas de las PcD y sus familias para enfrentar las condiciones sociales, económicas o ambientales que influyen en la salud.	N / MPV	8	18,6
		AV	7	16,3
		CS / S	28	65,1
	[Atención Médica] Entrega oportuna de atención para identificar, evaluar y/o tratar condiciones de salud de las PcD y sus familias.	N / MPV	1	2,3
		AV	3	7,0
		CS / S	39	90,7
	[Rehabilitación] Facilitar el logro de un funcionamiento óptimo de las PcD en su entorno.	AV	2	4,7
		CS / S	41	95,3
	[Dispositivos de Asistencia] Apoyo en el uso de dispositivos para asistir a una PcD.	AV	1	2,3
		CS / S	42	97,7
Educación	[Infancia Temprana] Apoyo a la inclusión de niños(as) con discapacidad en educación preescolar	N / MPV	34	79,1
		AV	7	16,3
		CS / S	2	4,7
	[Primaria] Apoyo a la inclusión de niños(as) con discapacidad en escuelas primarias.	N / MPV	35	81,4
		AV	6	14,0
		CS / S	2	4,7
	[Secundaria] Apoyo a la inclusión de jóvenes con discapacidad en la educación secundaria.	N / MPV	33	76,7
		AV	9	20,9
		CS / S	1	2,3
	[Superior] Apoyo al ingreso y mantención de jóvenes con discapacidad en la educación superior.	N / MPV	34	79,1
		AV	7	16,3
		CS / S	2	4,7
	[Educación no formal] Actividades educativas no formales para niños(as) y jóvenes con discapacidad en la comunidad	N / MPV	36	83,7
		AV	7	16,3
	[Aprendizaje de toda la vida] Actividades de aprendizaje para PcD que no han sido cubiertas por la educación formal	N / MPV	24	55,8
		AV	16	37,2
		CS / S	3	7,0
Subsistencia	[Desarrollo de Destrezas Básicas] Destrezas para el trabajo (alfabetización, habilidades matemáticas o para aprender)	N / MPV	21	48,8
		AV	14	32,6
		CS / S	8	18,6
	[Desarrollo de Destrezas Técnicas] Destrezas técnicas y profesionales (carpintería, zapatería, tejido, artesanía)	N / MPV	21	48,8
		AV	10	23,3
		CS / S	12	27,9
	[Desarrollo de Destrezas de emprendimiento] Destrezas para el emprendimiento (administración, planificación o trabajo con personas)	N / MPV	24	55,8
		AV	11	25,6
		CS / S	8	18,6
	[Desarrollo de Destrezas] Destrezas para la vida (trabajo en equipo, habilidades interpersonales, pensamiento creativo.	N / MPV	14	32,6
		AV	12	27,9
		CS / S	17	39,5
	[Trabajo por cuenta propia] Apoyo al desarrollo de actividades generadoras de ingresos por cuenta propia.	N / MPV	20	46,5
		AV	11	25,6
		CS / S	12	27,9
	[Trabajo Remunerado] Actividades para la superación y/o disminución de las barreras de ingreso al trabajo remunerado.	N / MPV	22	51,2
		AV	13	30,2
		CS / S	8	18,6
	[Protección Social, beneficios sociales] Promover el acceso a beneficios sociales entregados por el Estado u otras organizaciones.	N / MPV	9	20,9
		AV	12	27,9
		CS / S	22	51,2
	[Protección Social, redes de apoyo] Desarrollo de redes de apoyo social (grupos de autoayuda, organizaciones comunitarias, apoyo familiar).	N / MPV	6	14,0
		AV	6	14,0
		CS / S	31	72,1

Fuente: Elaboración propia.

N = nunca, MPV= muy pocas veces, AV= a veces, CS= Casi siempre, S= siempre, PcD = persona con discapacidad

El nivel de implementación de todas las actividades del componente Educación fue bajo, destacando con un 7% la actividad de “Aprendizaje de toda la vida”, en contraste con la ausencia de implementación de “Educación no formal” para niños(as) y jóvenes (Tabla 1).

Las actividades “Protección social, redes de apoyo” fueron las más realizadas en el componente Educación, con un 72,1%, mientras que “Desarrollo de destrezas básicas”, “Desarrollo de destrezas para el emprendimiento” y “Trabajo remunerado” se implementaron en un 18,6% cada una (Tabla 1).

Por otro lado, la mayoría de las actividades de los componentes Social y Fortalecimiento tuvo altos niveles de implementación (Tabla 2).

**Tabla 2:** Frecuencia de actividades realizadas componentes Social y Fortalecimiento. Centros Comunitarios de Rehabilitación, Región Metropolitana, Chile, 2016.

RBC	Preguntas	Categoría	n	%
Social	[Asistencia Personal] Apoyar el acceso y administración de la asistencia personal no familiar necesaria para vivir con autodeterminación y dignidad	N / MPV	12	27,9
		AV	16	37,2
		CS / S	15	34,9
	[Maternidad y Paternidad] Facilitar al acceso a servicios y programas para apoyar a PcD en su maternidad y paternidad	N / MPV	28	65,1
		AV	7	16,3
		CS / S	8	18,6
	[Relaciones, matrimonio y familia] Apoyo al desarrollo de relaciones satisfactorias con la familia.	N / MPV	7	16,3
		AV	10	23,3
		CS / S	26	60,5
	[Relaciones sociales] Incentivo y apoyo a PcD para que socialicen y desarrollen relaciones fuera del hogar.	N / MPV	2	4,7
		AV	7	16,3
		CS / S	34	79,1
	[Relaciones, matrimonio y familia] Sensibilizar sobre la discapacidad, prevenir violencia asociada y cambiar actitudes negativas tanto de familiares como de la comunidad.	N / MPV	5	11,6
		AV	5	11,6
		CS / S	33	76,7
	[Cultura y artes] Actividades que ayudan a desarrollar la identidad de las PcD	N / MPV	7	16,3
		AV	6	14
		CS / S	30	69,8
	[Cultura y artes] Desarrollar el sentido de pertenencia de PcD con su comunidad y territorio	N / MPV	9	20,9
		AV	8	18,6
		CS / S	26	60,5
	[Cultura y artes] Apoyar el acceso e inclusión de PcD a actividades culturales y artísticas	N / MPV	11	25,6
		AV	9	20,9
		CS / S	23	53,5
	[Recreación, ocio y deportes] Promover la participación de PcD en actividades de recreación y deportivas.	N / MPV	5	11,6
		AV	12	27,9
		CS / S	26	60,5
	[Justicia] Promover a PcD y familiares en el uso de apoyo comunitario y/o judicial cuando sus derechos son vulnerados.	N / MPV	10	23,3
		AV	16	37,2
		CS / S	17	39,5
Fortalecimiento	[Defensa y Comunicación] Incentivar el uso de tecnologías o apoyo para asegurar la accesibilidad de información y comunicación.	N / MPV	14	32,6
		AV	17	39,5
		CS / S	12	27,9
	[Defensa y representación] Apoyo a PcD y sus familias para que se representen a sí mismas en sus respectivas comunidades.	N / MPV	12	27,9
		AV	18	41,9
		CS / S	13	30,2
	[Movilización Comunal] Incentivar a la comunidad a mejorar la calidad de vida de las PcD y sus familias.	N / MPV	8	18,6
		AV	9	20,9
		CS / S	26	60,5
	[Participación Política] Promover la participación de las PcD en la política formal (toma de decisiones políticas y programas de desarrollo local o central)	N / MPV	15	34,9
		AV	12	27,9
		CS / S	16	37,2
	[Destrezas para la Participación Política] Apoyar a PcD y sus familias al acceso de información y desarrollo de destrezas que permitan participar en política	N / MPV	22	51,2
		AV	12	27,9
		CS / S	9	20,9
	[Grupos de Autoayuda] Incentivar grupos de autoayuda para realizar actividades y resolver problemas comunes.	N / MPV	13	30,2
		AV	9	20,9
		CS / S	21	48,8
	[Formación Organizaciones de PcD] Apoyar la formación de organizaciones de PcD	N / MPV	11	25,6
		AV	9	20,9
		CS / S	23	53,5

Fuente: Elaboración propia.

N = nunca, MPV= muy pocas veces, AV= a veces, CS= Casi siempre, S= siempre, PcD = persona con discapacidad’

La actividad “Relaciones sociales” tuvo el mayor porcentaje de realización (79,1%) del componente Social versus la actividad de “Maternidad y Paternidad” con un 18,6%.

Lo más desarrollado del componente Fortalecimiento fue la “Movilización comunal” (60,5%) en contraste con la actividad “Destrezas para la participación política” (20,9%).

Se elaboró un índice de implementación basado en los resultados de las actividades por cada uno de los componentes (Figura 2). Se asumió un nivel 100% de implementación a un componente en el cual las respuestas a todas las actividades fueron “Siempre” y 0% cuando las respuestas a todas las actividades fueron “Nunca”. Con base en dicho índice, se describió un nivel global de implementación de 61,5%, con un rango de 26,7% a 92,0%. El componente Educación presentó menor nivel de implementación, con una mediana de 30,0%, y un rango entre 20,0% y 63,3%, mientras que el componente Salud presentó mayor nivel de implementación con una mediana de 80,0% y un rango entre 45,7% a 100%. Los componentes Social, Fortalecimiento y Subsistencia presentan amplia dispersión de datos (variando entre un 20,0% y un 100% de implementación) y con valores medianos de 60, 72 y 62,9, respectivamente. Se identificó una correlación de dicho índice entre todos los componentes, excepto entre Educación y Salud (Rho de Spearman 0,298; p = 0,06), mientras que los niveles más altos de asociación en el nivel de implementación fue entre los índices Fortalecimiento y Social (Rho de Spearman 0,693; p < 0,001) y Salud y Subsistencia (Rho de Spearman 0,614; p < 0,001).

**Figura 2:** Índice de implementación por componente RBC. MeG = Mediana Global.

Fuente: Elaboración propia

Al comparar el nivel de implementación global y por componente de las actividades realizadas en los centros, aquellos que declaran realizar “Formación de organizaciones de personas con discapacidad” presentaron mayor nivel de implementación de la matriz de RBC que aquellos que no declaran realizarlas (p < 0,001 entre cada uno de los componentes).

Los equipos de trabajo contaron con distintos niveles de capacitación en los componentes de la matriz de RBC. Un 65,1% de los centros reportó un nivel “muy alto” y “alto” de capacitación en el componente Salud, mientras que un 62,8% de las respuestas en el componente Educación se distribuyeron en los niveles de capacitación “bajo” y “muy bajo” (Tabla 3).

**Tabla 3:** Percepción del nivel de capacitación del equipo profesional en los componentes de la RBC. Centros Comunitarios de Rehabilitación, Región Metropolitana, Chile, 2016.

Percepción del nivel global de capacitación		n	%
en salud	Muy Bajo	1	2,3
	Bajo	1	2,3
	Medio	13	30,2
	Alto	20	46,5
	Muy alta	8	18,6
en educación	Muy Bajo	9	20,9
	Bajo	18	41,9
	Medio	14	32,6
	Alto	2	4,7
	Muy alta	0	0,0
en subsistencia	Muy Bajo	5	11,6
	Bajo	7	16,3
	Medio	20	46,5
	Alto	8	18,6
	Muy alta	3	7,0
en componente social	Muy Bajo	3	7,0
	Bajo	11	25,6
	Medio	17	39,5
	Alto	8	18,6
	Muy alta	4	9,3
en fortalecimiento	Muy Bajo	3	7,0
	Bajo	11	25,6
	Medio	18	41,9
	Alto	5	11,6
	Muy alta	6	14,0

Fuente: Elaboración propia

RBC = Rehabilitación Basada en la Comunidad

Las principales fuentes de financiamiento de la RBC correspondieron al Ministerio de Salud (86,0%) y a los gobiernos locales (65,1%). También se reportaron otras fuentes de financiamiento, tales como fondos autogestionados (16,3%) y fondos aportados por el Servicio Nacional de Discapacidad (7,0%). Solo un centro reportó recibir financiamiento de fondos privados.

Los centros de rehabilitación comunitaria se vincularon con organizaciones comunitarias e instituciones del territorio comunal. Los organismos comunitarios y comunales con los que existía mayor vinculación fueron el gobierno local municipal (51,2%), las juntas de vecinos (37,2%), la Oficina Municipal de Discapacidad y Centros de Salud (ambos con 34,9%). Por otro lado, el gobierno central (14,0%), centros educacionales (14,0%), organizaciones de usuarios de salud (14,0%), fundaciones, corporaciones y organizaciones no gubernamentales (11,6%), Oficina Municipal de Intermediación Laboral (11,6%), organizaciones de personas con discapacidad (7,0%) y sector privado (4,7%), fueron los organismos con los que se reportó menor vinculación.

## DISCUSIÓN

Este estudio es una de las primeras aproximaciones a la evaluación de la implementación de la RBC en Latinoamérica tras la ratificación de la CDPD.

Se identificó un estado heterogéneo de implementación de la RBC en los centros comunitarios de rehabilitación de la Región Metropolitana de Chile, específicamente en lo relativo a los componentes de la matriz definida por la OMS. La implementación de la estrategia se centra en el componente “Salud”. Este componente es el más presente en las prácticas habituales de atención médica, rehabilitación y apoyo en el uso de dispositivos de asistencia y donde se reporta un mayor nivel de capacitación de los profesionales. Por el contrario, el componente “Educación” es el que reporta un menor nivel de implementación y de capacitación de los profesionales. Esto es consistente con el hecho de que las actividades e indicadores del programa de rehabilitación comunitaria del Ministerio de Salud chileno se centran en la rehabilitación física y en personas adultas como población objetivo^14^.

El principal objetivo de la estrategia de RBC es asegurar el máximo grado de inclusión social y ejercicio de derechos de las personas con discapacidad^3^. En Chile, se estableció como meta del programa RBC que un 10% de las personas atendidas en los centros de rehabilitación comunitaria logren la inclusión social (incorporación al trabajo, escuela, grupos sociales u organizaciones)^4^. Sin embargo, la mayoría de los centros reporta la realización de escasas actividades de los componentes de Educación y Subsistencia, como actividades de nivelación de estudios y desarrollo de destrezas laborales. Esto representa una barrera para el logro de los objetivos propuestos en el ámbito de la inclusión social y el ejercicio de los derechos. Este hallazgo es coincidente con otros estudios que evidencian que la incorporación de apoyos socio-económicos, tales como intermediación laboral o entrenamiento vocacional, adicionales a la rehabilitación física, contribuyen a la inclusión, a mejorar la autoestima de las personas y al cambio de actitud de la sociedad frente a ellas^15^.

Todos los centros cuentan con kinesiólogos/fisioterapeutas y terapeutas ocupacionales, y menos de la mitad cuenta con profesionales de otras disciplinas, tales como fonoaudiólogos/logopedas, enfermeras(os), psicólogos y técnicos de nivel superior. Si bien la existencia de la dupla kinesiólogo/fisioterapeuta - terapeuta ocupacional es un avance en la implementación de un modelo de rehabilitación integral, sigue siendo insuficiente para una adecuada implementación de la estrategia RBC. A esto se suma la poca presencia de trabajo intersectorial. El 51,2% de los centros reporta vincularse con el gobierno local en el contexto de la Región Metropolitana. Además, es escasa la participación de otros actores, tales como monitores y agentes comunitarios, que promuevan la realización de actividades de prevención primaria y secundaria y otras actividades relacionadas con la salud mental, la educación y el fortalecimiento comunitario. Este hallazgo coincide con estudios de evaluación de programas de RBC realizados en otros países (Malawi, Kenia, Tanzania y Uganda) que indican que la escasez de recursos humanos y de capacitación es una limitación importante para su implementación^16^.

Si esperamos cumplir con “no dejar a nadie atrás, a través de la completa implementación de la Convención sobre los Derechos de las Personas con Discapacidad”, como estableció el comité de seguimiento de la CDPD en junio de 2018, debemos posicionar la RBC como una estrategia intersectorial que logre la inclusión social. Seguir enfocados en solo reducir la brecha de acceso a rehabilitación no dará ni las herramientas ni los resultados requeridos para el desarrollo sostenible a alcanzar al 2030.

Se reconoce como limitaciones de este estudio que la información fue obtenida desde los coordinadores(as) de la RBC. Esto genera la posibilidad de sobreestimar el estado de implementación al dar cuenta de su propia gestión como responsables. No obstante, se requiere la perspectiva de los actores responsables de los centros de rehabilitación comunitaria, ya que en su rol de coordinación, tienen gran importancia en la implementación del programa. Futuras investigaciones deben incluir al equipo profesional y a las personas con discapacidad y sus familias en los procesos de evaluación de los centros comunitarios para profundizar y captar la complejidad de la implementación. Esto permitiría dar cumplimiento a los principios de la CDPD y a una evaluación integral de las actividades realizadas.

Además de eso, solo es posible referirse al estado de implementación de la RBC en la Región Metropolitana y no a todo el territorio nacional, dadas las características geográficas y sociodemográficas propias de la región en cuanto capital del país. Por otro lado, no existía un instrumento para evaluar las actividades realizadas en el contexto de la implementación de la RBC en Chile. A partir de este estudio, se puede disponer de un instrumento que permite el levantamiento de información completa sobre la implementación de la RBC en todo el territorio nacional.

La implementación de la RBC en Chile está centrada en el componente Salud de la matriz propuesta por la OMS. Este hecho debe ser tomado en cuenta en los procesos de evaluación y formulación de planes de mejora de la estrategia. No solo se debe continuar con la implementación de los demás componentes de la matriz, sino que completar la implementación del componente Salud. Esto a través de la incorporación de actividades de promoción, prevención, apoyo al uso de ayudas técnicas y atención integral que incluya atención en el ámbito de la salud mental para acompañar los procesos de aceptación y adaptación, tanto de las personas con discapacidad como de su familia.

La estrategia debe potenciar el trabajo intersectorial y reconocer a los actores institucionales y comunitarios, con quienes deben generar alianzas para sumar esfuerzos que busquen construir una sociedad más inclusiva. La RBC, incluso en aquellos programas centrados en salud, puede potenciar una positiva convivencia entre los modelos de comprensión de la discapacidad (médico y social). Esto, siempre y cuando las prestaciones de rehabilitación respondan a las necesidades e intereses de las personas con discapacidad y existan instancias de formación y reflexión en torno al rol de la sociedad en el establecimiento de barreras para la inclusión. Es necesario contar con mayor diversidad de profesionales que permitan ampliar las actividades que se realizan en los centros de RBC. Por otro lado, es esencial apoyar el desarrollo de organizaciones de personas con discapacidad para la defensa de sus derechos y así avanzar en la construcción de una sociedad más justa e inclusiva.
